# Palmitic Acid Upregulates Type I Interferon–Mediated Antiviral Response and Cholesterol Biosynthesis in Human Astrocytes

**DOI:** 10.1007/s12035-023-03366-z

**Published:** 2023-05-15

**Authors:** Alexis Felipe Rojas-Cruz, Cynthia Alexandra Martín-Jiménez, Janneth González, Yeimy González-Giraldo, Andrés Mauricio Pinzón, George E. Barreto, Andrés Felipe Aristizábal-Pachón

**Affiliations:** 1 Departamento de Nutrición Y Bioquímica, Facultad de Ciencias, Pontificia Universidad Javeriana, Bogotá, 110231 Colombia; 2 Department of Neuroscience and Regenerative Medicine, Medical College of Georgia at Augusta University, Augusta, GA 30912 USA; 3 Laboratorio de Bioinformática Y Biología de Sistemas, Universidad Nacional de Colombia, Bogotá, 110231 Colombia; 4 Department of Biological Sciences, University of Limerick, V94 T9PX Limerick, Ireland

**Keywords:** Human astrocytes, Lipotoxicity, Tibolone, RNA-seq, Neuroinflammation, Lipid metabolism

## Abstract

Chronic intake of a high-fat diet increases saturated fatty acids in the brain causing the progression of neurodegenerative diseases. Palmitic acid is a free fatty acid abundant in the diet that at high concentrations may penetrate the blood–brain barrier and stimulate the production of pro-inflammatory cytokines, leading to inflammation in astrocytes. The use of the synthetic neurosteroid tibolone in protection against fatty acid toxicity is emerging, but its transcriptional effects on palmitic acid–induced lipotoxicity remain unclear. Herein, we performed a transcriptome profiling of normal human astrocytes to investigate the molecular mechanisms by which palmitic acid causes cellular damage to astrocytes, and whether tibolone could reverse its detrimental effects. Astrocytes undergo a profound transcriptional change at 2 mM palmitic acid, affecting the expression of 739 genes, 366 upregulated and 373 downregulated. However, tibolone at 10 nM does not entirely reverse palmitic acid effects. Additionally, the protein–protein interaction reveals two novel gene clustering modules. The first module involves astrocyte defense responses by upregulation of pathways associated with antiviral innate immunity, and the second is linked to lipid metabolism. Our data suggest that activation of viral response signaling pathways might be so far, the initial molecular mechanism of astrocytes in response to a lipotoxic insult by palmitic acid, triggered particularly upon increased expression levels of *IFIT2*, *IRF1*, and *XAF1*. Therefore, this novel approach using a global gene expression analysis may shed light on the pleiotropic effects of palmitic acid on astrocytes, and provide a basis for future studies addressed to elucidate these responses in neurodegenerative conditions, which is highly valuable for the design of therapeutic strategies.

**Supplementary Information:**

The online version contains supplementary material available at 10.1007/s12035-023-03366-z.

## Introduction

Astrocytes are located in the entire central nervous system (CNS), playing different roles such as regulation of homeostasis and synapses; they participate in inflammatory processes and contribute to the maintenance of the blood–brain barrier (BBB), among others. Due to these important functions, astrocytes have been related to the development of several diseases such as neurodegenerative and neuropsychiatric disorders (ND) [1]. Astrocytes respond to stressful conditions to maintain and protect brain functions through a mechanism named reactivity [2]. However, when these cells become reactive, they not only can trigger protective effects, but also induce detrimental actions, which depend on specific contexts such as diseases, infections, injuries, and others [3]. It should be noted that astrocytes are heterogeneous, and they have different molecular, morphological, and functional profiles depending on the brain region [4], and most important, mouse and human astrocytes trigger different signals under stressful conditions [5].

In order to know the possible role of astrocytes in the development of ND, a large number of studies have analyzed the effects of several molecules in in vitro and in vivo models [6–9]. Nowadays, several studies are focused on assessing the effects of palmitic acid (PA), a saturated fatty acid that is part of cell membranes and is involved in several processes such as protein palmitoylation and palmitoylethanolamide synthesis [10]. The interest in this fatty acid has arisen due to high levels of PA trigger detrimental effects on the brain. For instance, it can induce the production of pro-inflammatory cytokines, reactive oxygen species (ROS), and endoplasmic reticulum stress, altering the functions of different types of cells [11]. Moreover, it has been reported an increased concentration of PA in the brains of Alzheimer’s disease (AD) patients [12]. These findings suggest that PA could be involved in the pathophysiological mechanism of ND [13], and thus, it has been used to evaluate the protective effects of different drugs, including hormones [14].

On the other hand, previous studies have found that tibolone (TIB), a synthetic steroid with estrogenic, progestogenic, and androgenic actions, protects astrocytes against the detrimental effects induced by PA. For instance, TIB attenuates inflammatory responses induced by PA [15]; it reduces oxidative damage and preserves mitochondrial functionality [16–18]. Although some genes have been explored in astrocytic cell models submitted to PA and TIB stimuli [15], to the best of our knowledge, there is no evidence of studies analyzing transcriptome-wide expression under these conditions. Regarding downstream molecules, such as proteins and metabolites, two recent studies have investigated the effects of PA and TIB treatments in in vitro experiments of astrocytes [19, 20].

Considering the relevance of PA to elucidate the role of astrocytes in relation to ND, this study analyzes the transcriptome profile of normal human astrocytes (NHA) to investigate the molecular mechanisms of lipotoxic-triggering stimuli by PA, and whether TIB might reverse its detrimental effects, according to conditions previously standardized by [18]. Interestingly, we found that PA may activate relevant genes associated with astrocyte defense responses by upregulating pathways associated with antiviral innate immunity and lipid metabolism. On the contrary, TIB has no effect in ameliorating PA-induced transcriptomic changes.

## Materials and Methods

### Cells and Culture Methods

NHA cells were obtained from Lonza (Basel, Switzerland, Catalog: CC-2565). Three different batches of NHA cells: (i) 0000514417 (male donor), (ii) 00005656712 (female donor), and (iii) 0000612736 (female donor) were grown at 37 °C with a humidified atmosphere and 5% CO_2_. The cells were seeded at 5000 cells/cm^2^ and kept for 12 days until they reached 80% of confluence using the astrocyte basal medium (ABM) (Lonza; Basel, Switzerland) supplemented with SingleQuots supplements (Lonza; Basel, Switzerland).

### Palmitic Acid and Tibolone Treatments

The experimental model employed in the current work was previously described by [18]. Briefly, the supplemented ABM was changed to serum-free Dulbecco’s modified eagle medium (DMEM) without phenol red (Lonza; Basel, Switzerland). After 6 h of serum starvation, TIB (Sigma; St. Louis, MO, USA) (Lot: T0827) was added to NHA cells at 10 nM for 24 h. Once this was completed, the cells were treated with 2 mM PA (Sigma; St. Louis, MO, USA) for 24 h; PA alone causes a 50% cytotoxicity in PI uptake (Fig. S1a). The cells were washed with 1 × phosphate-buffered saline (PBS) before adding each treatment. Time and concentrations implemented in this study were selected considering the effect of TIB attenuates 53% of cell death induced by PA (Fig. S1b). Each treatment was carried out for duplicate using the three different batches of NHA cells.

The controls for these experimental conditions were the following: (i) untreated cells (DMEM) were used as a control for TIB treatment; and (ii) a vehicle (VH) containing 1.35% bovine serum albumin (BSA) (Sigma; St Louis, MO, USA), and 2 mM carnitine (Sigma; St Louis, MO, USA) was considered as a control for PA treatment, and the combination of TIB plus PA.

### RNA Isolation, Library Preparation, and RNA Sequencing

The total RNA was isolated from 30 samples of cultured astrocytes, namely from the 3 treatments (PA, TIBPA, TIB), alongside the 2 controls (DMEM, VH), which were carried out in 3 biological replicates per duplicate, using RNeasy mini kit (Qiagen; Hilden, Germany) according to the manufacturer’s recommendations (Table S1). To remove possible DNA contamination, samples were treated with DNase I (New England Biolabs, Ipswich, MA, USA). The quantity and quality of RNA were assessed using the NanoDrop 2000 (Thermo Fisher Scientific, Waltham, MA, 174 USA) and the Agilent 2100 bioanalyzer (Agilent Technologies, Palo Alto, Calif.), respectively. Samples with an RNA integrity number (RIN) ≥ 8.2 were taken forward to perform a strand-specific (antisense) RNA library preparation with poly (A) + selection using the TruSeq stranded mRNA library preparation kit following the Illumina manufacturer’s protocol (San Diego, CA, USA) (Catalog: RS-122–2101). We used the Illumina HiSeq sequencing platform to generate 150-bp paired-end sequencing reads.

### RNA-seq Data Analysis

We analyzed the RNA-seq data from astrocytes with the “rnsaseq” (v3.6) pipeline implemented in nf-core [21]. An initial quality check of sequenced libraries was undertaken using FastQC (v0.11.9) and Trim Galore (v3.4). To remove ribosomal RNA (rRNA), clean reads were sorted into rRNA and non-rRNA using SortMeRNA (v4.3.4) [22] with eight references including Rfam [23] and SILVA [24] (Table S2). The non-rRNA reads were mapped on the human reference genome obtained from GENCODE (HG38-Release 37 [GRCh38.p13]) using the Spliced Transcripts Alignment to a Reference (STAR) package (version 2.7.6a) [25], with the standard parameters for paired reads. The quantification of expressed genes for each sequenced sample was computed using RNA-Seq by expectation–maximization (RSEM) (v1.3.3) [26]. The RSeQC package (v3.0.1) [27] was used to assess the quality of sequencing depth and alignments in order to identify possible biases and batches in our samples.

### Differential Gene Expression Analyses

Differential expression (DE) analysis was performed from gene-level counts using the edgeR package (v3.36) in R [28]. The identification of differentially expressed genes (DEGs) was performed by contrasting: (i) PA and TIBPA with VH, (ii) TIBPA with PA, and (iii) TIB with DMEM. We first performed a pre-filtering to discard genes with read counts less than 2 across all samples, and then genes with a minimum requirement of 20 counts per million (CPM) across libraries for each group were kept. Gene counts were normalized for RNA composition among libraries using a trimmed mean of *M*-values (TMM) normalization [29]. To corroborate the read count normalization, we used boxplot, and principal component analyses (PCA) (Fig. S2). The comparisons were tested with a quasi-likelihood *F*-test [29], which accounts for the uncertainty in dispersion estimation at the groups of PA, TIBPA, and TIB, separately. For each comparison, genes with |log2 (fold change, FC)|> 1 and false discovery rate (FDR) adjusted *p-*value < 0.05 using Benjamini–Hochberg’s procedure were considered DEGs. To uncover which DEGs are shared across the contrasts of each PA, TIBPA, and TIB treatment group, we plotted Venn diagrams with the ggVennDiagram package (v2.1) [30] implemented in R. The identification of overlapping DEGs was conducted by differentiating those that were significantly up- and downregulated.

### Functional Enrichment Analysis

Functional analysis was carried out to identify gene sets of interest for up- and downregulated DEGs using the compareCluster function of the clusterProfiler package (v4.0.5) [31] in R. The over-representation analysis (ORA) based on hypergeometric distribution with additional multiple hypothesis-testing corrections was run with the overlapping DEGs through a formula interface: *Entrez ∼ treatment* + *regulation.* We performed Gene Ontology (GO) terms (Biological Process [BP], Cellular Component [CC], and Molecular Functions [MF]) [32], using the “enrichGO” function. To identify the most important pathways mediating the different transcriptional regimes, we used the function “enrichKEGG” to perform the Kyoto Encyclopedia of Genes and Genomes (KEGG) [33] pathway enrichment. We confirmed the results of the KEGG enrichment using the Reactome database [34], with the function “enrichPathway”. FDR-adjusted *p*-value < 0.05 using the Benjamini–Hochberg correction method was applied for all enrichment analyses. In addition, we generated GOChords representations of the 8 most representative BP terms for the treatments, and GOBubbles to gain a full understanding of GO terms using the GOplot package (v1.0.2) [35]. GO term results were confirmed with further tools including Protein ANalysis THrough Evolutionary Relationships (PANTHER) (v14.0) [36], and the Database for Annotation, Visualization and Integrated Discovery (DAVID) (release: May 15, 2022) [37].

### Establishment of a Protein–Protein Interaction Network and Identification of Hub Genes

The up- and downregulated DEGs in GO terms were employed to establish the protein–protein interaction (PPI) network. PPI network was constructed from the STRING database (v11.5) [38] using Cytoscape (v3.9.1) [39] via stringApp (v1.7.0) [40]. The PPI score set as 0.9 (high confidence) was considered significant. The molecular complex detection (MCODE) plug-in (v2.0.0) [41] of the Cytoscape tool was applied to find significant gene clustering modules, as default, with degree cutoff = 2, node score cutoff = 0.2, *k*-core = 2, and max.depth = 100. The hub genes were screened out by cytoHubba plug-in (v0.1) [42], which identifies hub genes through multiple algorithms. The intersections of four ranking algorithms, including maximum neighborhood component (MNC), degree, maximal clique centrality (MCC), and density of maximum neighborhood component (DMNC), were considered hub genes.

### Validation of Gene Expression by Real-time Quantitative Polymerase Chain Reaction

For this analysis, the three batches of NHA cells were treated with PA and VH as described above, and RNA was isolated from three technical replicates for each treatment. RNA was isolated using the RNeasy mini kit (Qiagen; Hilden, Germany) according to the manufacturer’s recommendations. These samples were quantified through a NanoDrop 2000 (Thermo Fisher Scientific, Waltham, MA, 174 USA), and treated with DNase I (New England Biolabs, Ipswich, MA, USA). Next, cDNA was generated using the OneScript® Plus cDNA Synthesis Kit (ABM, Richmond, Canada), employing 13 μL of RNA at 20 ng/μL, 0.5 μM Oligo(dT), 0.5 mM dNTP, 1 × RT buffer, and 1 μL of reverse transcriptase following the manufacturer’s recommendations. Before running the quantitative PCR (RT-qPCR), a dilution of 1:2 of the cDNA was done. RT-qPCR was performed with the SensiFAST™ SYBR No-ROX Kit (Bioline, London, UK), which was mixed with 1 µL of cDNA, 400 nM of each primer (forward and reverse), and water was added for a total volume of 10 µL. The PCR program consisted of a cycle of 95 °C for 10 min, 40 cycles of 95 °C for 15 s, and 60 °C for 60 s, followed by melting analysis. The annealing temperature and the primer sequence for each gene analyzed are described in Table S3. The relative gene expression was calculated using the comparative CT method [43], and for the normalization, the glyceraldehyde-3-phosphate dehydrogenase (*GAPDH*) gene was used as housekeeping gene for normalization. Statistical analysis was carried out in the GraphPad Prism (v8.0.1) software (GraphPad Software, La Jolla, CA, USA). An unpaired *t*-test was used to compare the treatments and a *p-*value < 0.05 was considered significant.

## Results

### Palmitic Acid–Induced Transcriptomic Changes in Human Astrocytes

**Fig. 1:**
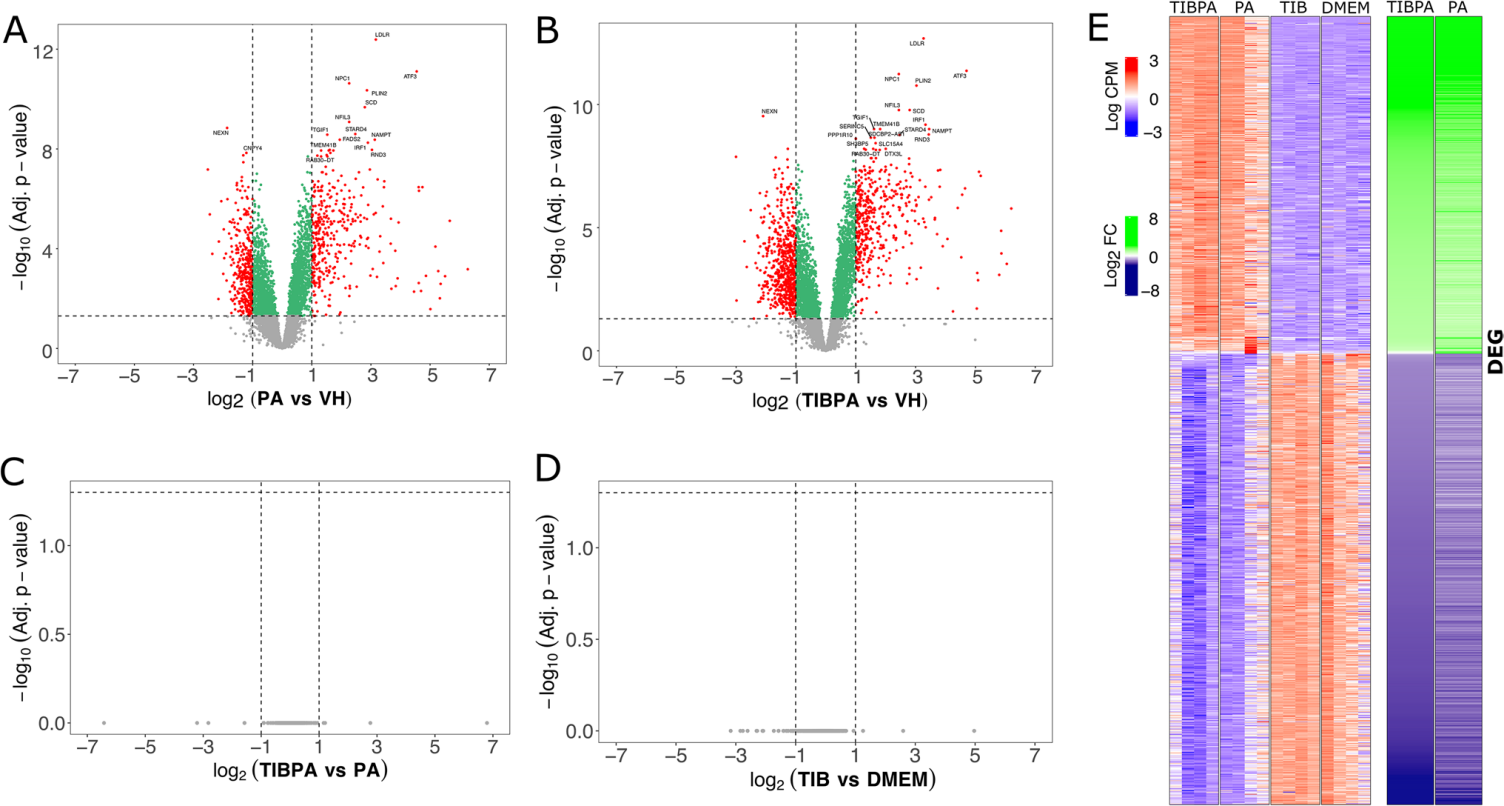
Transcriptomic profile of human astrocytes in response to treatments with PA, TIBPA, and TIB. Volcano plots showing the distribution of –log10 in FDR-corrected *p-*value and the log_2_FC of DEGs for **A**,** B** PA (PA vs VH) and (TIBPA vs VH), respectively. For **C**,** D** TIB (TIBPA vs. PA), and (TIB vs. DMEM), respectively. Each comparison includes quadruplicate samples for the treated conditions (PA, TIBPA, and TIB) and the untreated control (DMEM and VH). Statistically significant DEGs are shown as red dots with |log_2_FC|> 1 and FDR-adjusted *p-*value < 0.05 by Benjamini–Hochberg method. **E** Heatmap of DEGs showing the absent effect of TIBPA against PA, and TIB upon a comparison of DMEM. The left panel shows read counts per gene in log CPM (counts per million) rows for the indicated treatment conditions, while orange and purple colors denote higher and lower expression, respectively. The right panel shows differential expression in log_2_FC of genes upon TIBPA and PA conditions. Green and purple colors denote positive and negative differences in gene expression, respectively. PA, palmitic acid; TIBPA, tibolone + palmitic acid; TIB, tibolone; VH, vehicle; and DMEM, Dulbecco’s modified eagle medium

The viability of NHA is altered by 2 mM PA for 24 h (Fig. S1); at the transcriptional level, our analysis showed that this condition triggers a total of 694 DEGs, of which 350 and 344 were up- and downregulated, respectively (Fig. 1A). Similar to PA, TIBPA registered 1052 DEGs, showing 442 up- and 610 downregulated when compared with VH (Fig. 1B), suggesting that the treatment with TIBPA does not differ notably regarding PA. Next, we asked whether at 10 nM TIB might exert protective actions against PA toxicity. For this end, potential DEGs were sought following two scenarios: (i) we first compared TIBPA to induce changes in gene expression in contrast to PA; and (ii) then explored TIB with its baseline DMEM. Intriguingly, either in combination with PA (Fig. 1C) or TIB alone (Fig. 1D), we found that TIB failed to exacerbate PA-induced expression changes. Indeed, heatmap analysis showed that TIBPA with PA had similar expression levels compared between TIB and DMEM (Fig. 1E).

**Fig. 2:**
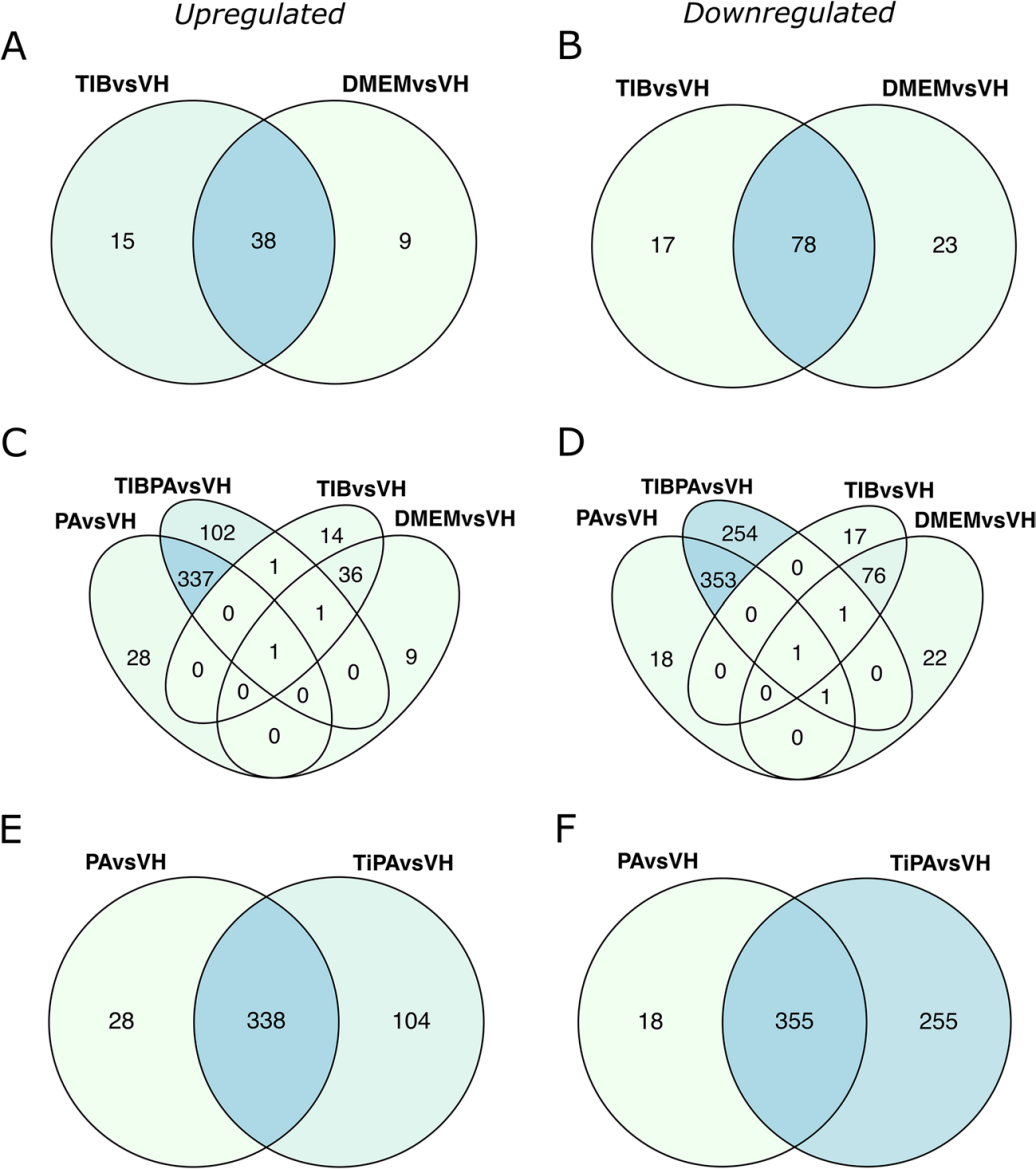
Cross-referencing analysis for identifying up- and downregulated DEGs in TIB and PA conditions.** A**,** B**,** C**, **D** Venn diagrams show the process to find potential DEGs in TIB by means of PA and TIBPA contrasts. **E**,** F** Venn diagrams registering the number of representatives up- and downregulated DEGs triggered by PA. Sections are colored based on DEG counts. PA, palmitic acid; TIBPA, tibolone + palmitic acid; TIB, tibolone; VH, vehicle; and DMEM, Dulbecco’s modified eagle medium

Although TIBPA with PA and TIB with DMEM did not show DEGs, the comparison between TIBPA and VH showed a higher number of DEGs than PA. Taking into account these results, we implemented the strategy of cross-referencing DEGs in order to explore whether TIB could be modulating the expression of specific genes. Therefore, further comparisons between TIB with VH, and DMEM with VH were performed. This resulted in 32 DEGs, where 15 were up- (Fig. 2A) and 17 downregulated (Fig. 2B). After that, we used PA and TIBPA with VH to explore whether these DEGs were unique to TIB, considering the presence of TIB. Interestingly, this analysis matched 1 upregulated DEG (Fig. 2C), namely dual specificity protein phosphatase 1 (*DUSP1*), which perhaps might be induced by TIB.

On the other hand, comparisons between PA and TIBPA were cross-referenced to establish a consistent list associated with PA-stimulated DEGs, given both treatments contain PA. Overlapping DEGs, along with the PA intercept were retrieved, identifying 739 DEGs. Among them, 366 were up- (Fig. 2E) and 373 downregulated DEGs (Fig. 2F). Full information on DEGs related to PA is available in Table S4.

### Palmitic Acid Alters the Expression of Miscellaneous Inflammatory Genes

**Fig. 3:**
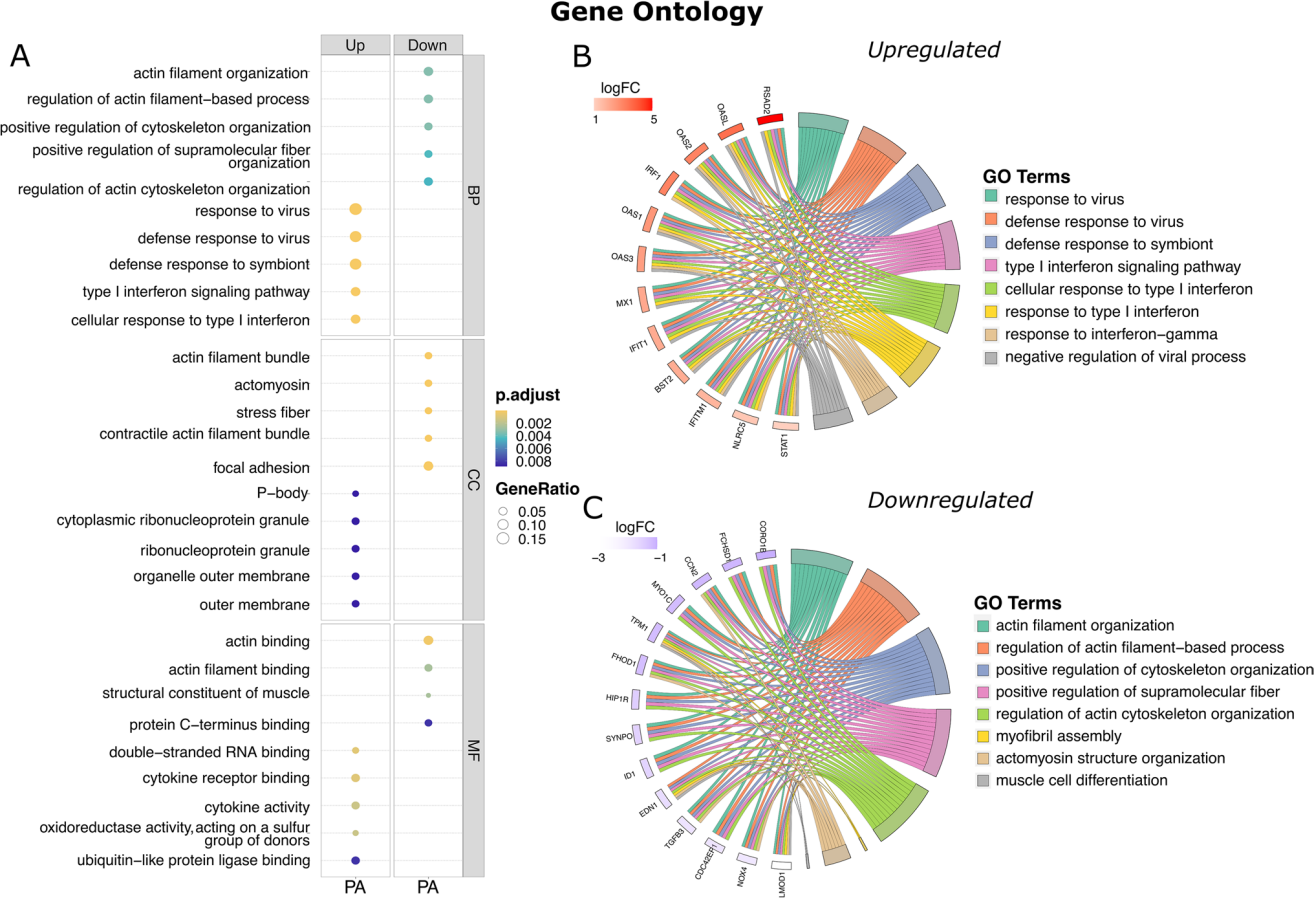
Comparison of functional enrichment analysis for up- and downregulated DEGs in astrocytes under PA stimulus. **A** Over-representation analysis for Gene Ontology showing categories of Biological Process (BP), Molecular Function (MF), and Cellular Component (CC) in the top DEGs. Circle size is proportional to the GeneRatio, while color denotes significance (orange is more significant, and purple is less significant). **B**,** C** GOchord plots showing the top eight over-represented GO BP terms for up- and downregulated DEGs related to PA. DEGs are linked to their assigned BP term by colored ribbons, and ordered according to the log_2_FC, which is displayed in descending intensity of red/purple squares. Significantly enriched GO terms were carried out considering FDR-adjusted *p-*value < 0.05 by Benjamini–Hochberg method

To better understand the functional alterations induced in human astrocytes following PA stimulation, we performed an ORA with clusterProfiler through the formula: *Entrez* ∼ *treatment* + *regulation*, comparing up- or downregulated DEGs. GO enrichment analysis showed that about 42% of enriched terms in the BP category for upregulated DEGs were associated with antiviral and inflammatory responses, whereas over 70% for downregulated DEGs were linked to regulated processes in actin filament organization (Fig. 3A). Notably, we found the three most enriched BP terms for upregulated DEGs, including (i) response to virus (GO:0009615), (ii) defense response to virus (GO:0051607), and (iii) defense response to symbiont (GO:0140546) (Fig. 3B and Fig. S3a), where the most over-expressed DEGs (log_2_FC = 5.66 to 5.36) comprised the cytokine family such as C-X-C motif chemokine ligand 10 (*CXCL10*), C-X-C motif chemokine ligand 9 (*CXCL9*), and C–C motif chemokine ligand 8 (*CCL8*). In contrast, downregulated DEGs ranged in three more enriched terms, as follows: (i) actin filament organization (GO:0007015), (ii) regulation of actin filament-based process (GO:0032970), and (iii) positive regulation of cytoskeleton organization (GO:0051495) (Fig. 3C and Fig. S3b), whose DEGs (log_2_FC =  − 2.52 to − 1.90) included Leiomodin 1 (*LMOD1*), Actin Alpha Cardiac Muscle 1 (*ACTC1*), and Refilin A (*RFLNA*) (Table S5).

**Fig. 4:**
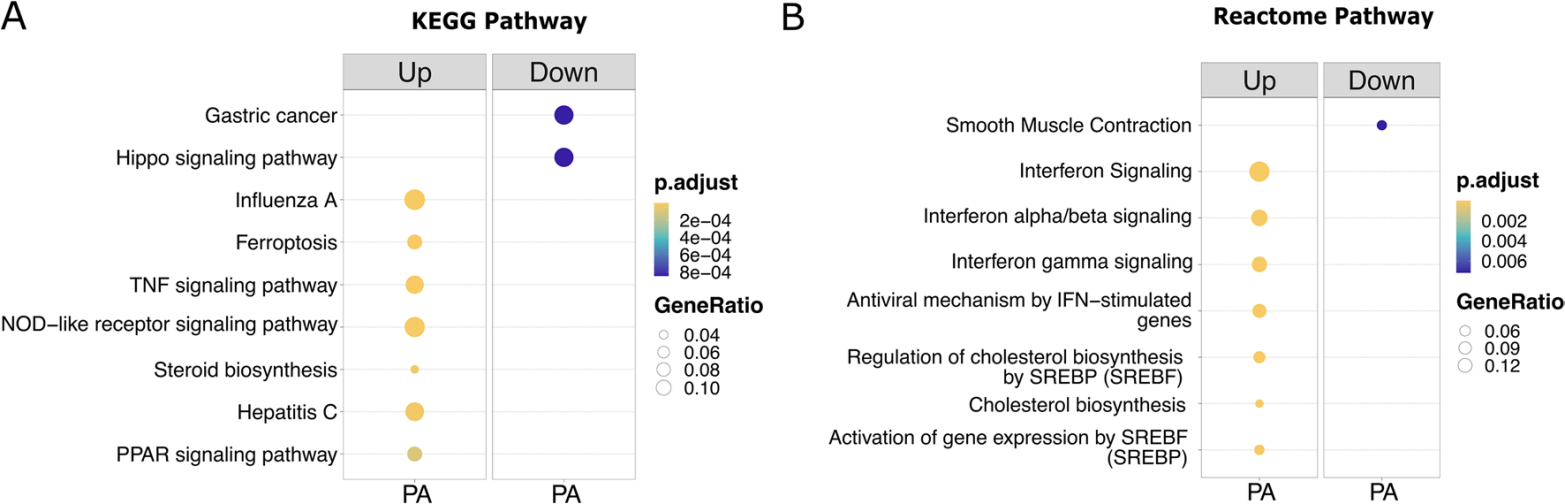
Comparison of enriched pathways for up- and downregulated DEGs in astrocytes under PA stimulus. **A**,** B** KEGG and Reactome analysis comparing the most enriched pathways in PA, respectively. Significantly enriched KEGG and Reactome analysis were carried out considering FDR-adjusted *p-*value < 0.05 by Benjamini–Hochberg method. Circle size is proportional to the GeneRatio, while color denotes significance (orange is more significant, and purple is less significant). PA, palmitic acid

KEGG and Reactome were used to elucidate the most enriched pathways in the dysregulated astrocytes. Even though diverse pathways were uncovered in the databases for upregulated DEGs, most of them hinted at two main processes: (i) antiviral innate immunity pathways; and (ii) lipid metabolism (Fig. 4). Among the antiviral innate immunity pathways, we found the tumor necrosis factor (TNF) signaling, NOD-like receptor (NLRs), and interferon (IFN) family, with cytokine *CXCL10* and pro-inflammatory genes such as interferon-induced protein with tetratricopeptide repeats 2 (*IFIT2*), and radical S-adenosyl methionine domain containing 2 (*RSAD2*), considered the most significant DEGs (log_2_FC = 5.66 to 4.61). Regarding the second most enriched pathway, lipid metabolism, we detected steroid and cholesterol biosynthesis as well as the peroxisome proliferator–activated receptor (PPAR) signaling pathway. In addition, DEGs involved in fat body induction (log_2_FC = 4.62 to 3.42), such as angiopoietin Like 4 (*ANGPTL4*), matrix metallopeptidase 1 (*MMP1*), and fatty acid binding protein 3 (*FABP3*) were highlighted. Collectively, the enrichment analyses suggest that PA may induce pro-inflammatory cytokine expression by upregulating pathways of antiviral innate immunity as well as genes related to lipid metabolism in human astrocytes.

### Palmitic Acid–Exposed Astrocyte Interactome Reveals Two Protein Clustering Modules

**Fig. 5:**
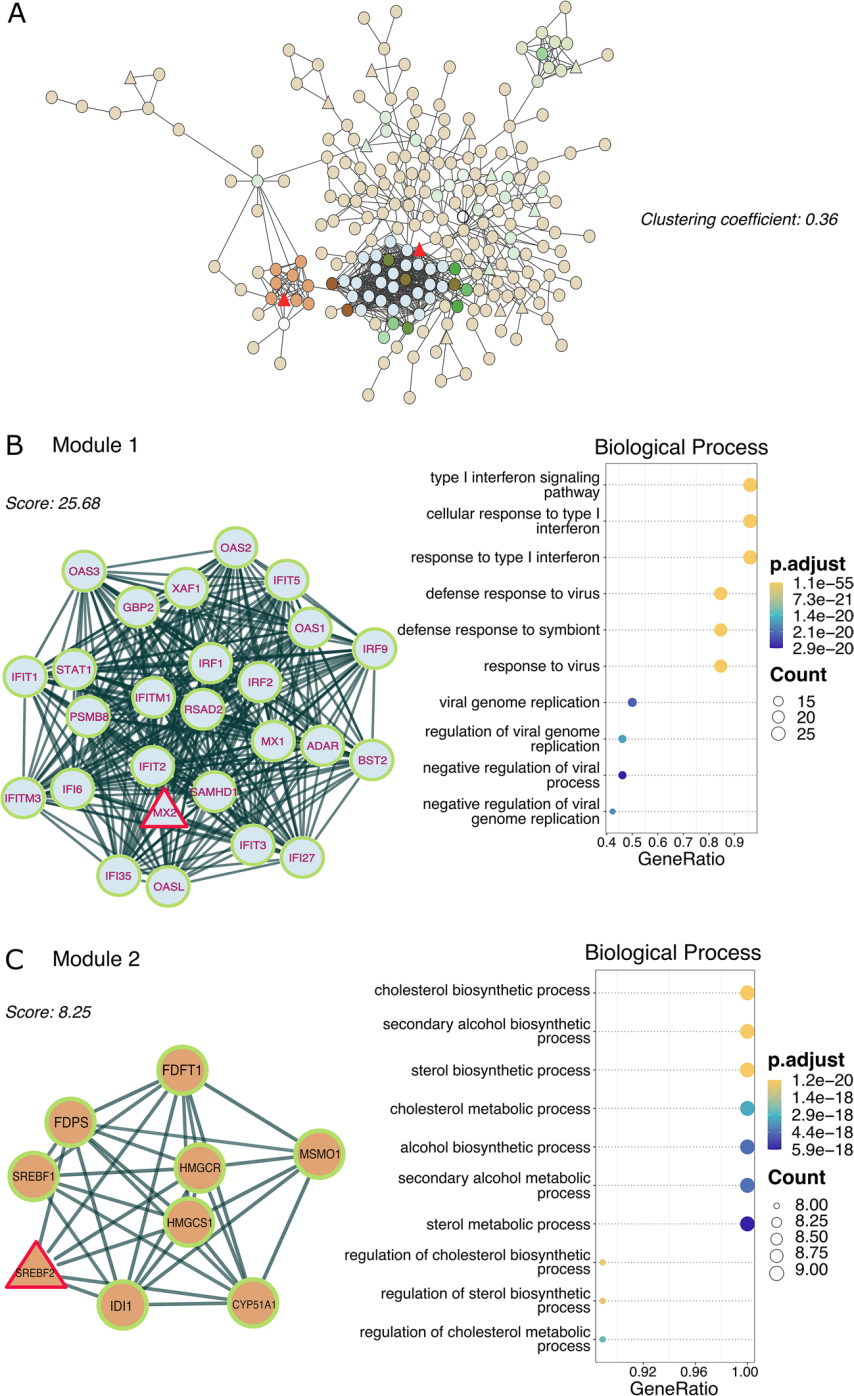
Astrocyte interactome and identification of hub genes upon PA-induced stress. **A** PPI network diagram construction based on DEGs (across BP, CC, and MF ontological categories) consisting of 778 edges and 417 nodes. The color of the nodes corresponds to their MCODE degree values. Nodes with a red triangular shape are the seed proteins, while the rest are their neighbors. **B** The first significant gene clustering module extracted by MCODE contained 321 edges and 26 nodes with modular gene enrichment analysis. **C** The second gene clustering module with 33 edges and 9 nodes, and their enrichment analyses. Adjusted *p-*value < 0.05 was considered significant and circle size is proportional to the gene count, while color denotes significance (orange is more significant, and purple is less significant)

Next, we proposed unraveling how these proteins interact during PA lipotoxic conditions. To this end, we concatenated up- and downregulated DEGs across all three ontological categories (BP, CC, and MF), and subsequently removed redundant proteins, retrieving 380 of them. The PPI network from STRING was composed of 778 edges and 417 nodes, with an average local clustering coefficient of 0.36 (Fig. 5A). Additionally, we used the MCODE plug-in to investigate whether PA-associated proteins might form a highly connected molecular module. The MCODE clustering analysis identified two densely connected modules, with members ranging from 9 to 26 proteins. Module 1 was the most interconnected cluster (MCODE score = 25.68) with 321 edges and 26 nodes, identifying the upregulation of MX dynamin-like GTPase 2 (*MX2*) as the seed protein (Fig. 5B). In addition, GO BP and KEGG analysis showed that proteins were related to antiviral and inflammatory responses, triggering primarily antiviral innate immunity pathways (Fig. S4a). Conversely, module 2 (MCODE score = 8.25) showed fewer proteins and consisted of 33 edges and 9 nodes, with the over-expression of sterol regulatory element binding transcription factor 2 (*SREBF2*) as seed protein (Fig. 5C). Based on GO enrichment, the BP acted primarily on steroid and cholesterol biosynthesis; claimed through KEGG to be involved in lipid metabolism pathways (Fig. S4b). Interestingly, the PPI network reveals two protein clustering modules that are consistent with previous enrichment analyses (Fig. 4), suggesting that PA is potentially causing astrocytes to activate antiviral innate immunity, and lipid metabolism pathways. A detailed functional enrichment report for the two identified modules in the PPI network is provided in Table S6.

### Identification of Hub Genes and Validation of Expression Analysis by qPCR

**Fig. 6:**
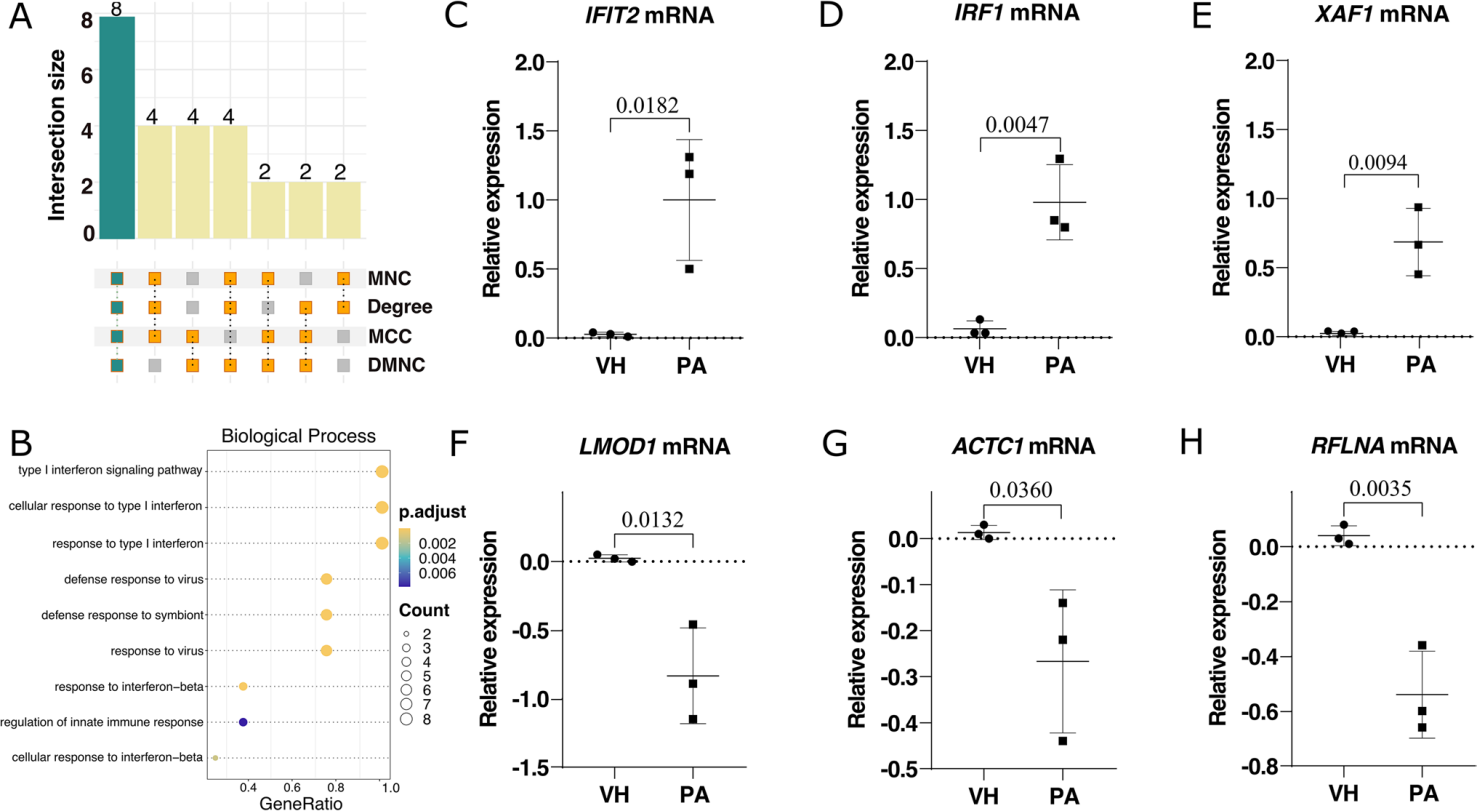
Common hub genes and validation of human astrocyte gene expression by qPCR upon PA toxicity. **A** The UpSet diagram showed 8 overlapping hub genes screened by four different algorithms (MNC, Degree, MCC, and DMNC) with the Cytoscape plug-in cytoHubba. The aquamarine squares denote when the four algorithms match at least one gene, the yellow squares show matches between two or three algorithms, and the gray squares reflect mismatches. **B** Gene enrichment analysis of hub genes. Adjusted *p-*value < 0.05 was considered significant and circle size is proportional to the gene count, while color denotes significance (orange is more significant, and purple is less significant). **C**,** D**, **E** Relative expression of the top 3 upregulated genes (*IFIT2*, *IRF1*, and *XAF1*) based on hub genes, and **F**,** G**, **H** the top 3 downregulated genes (*LMOD1*, *ACTC1*, and *RFLNA*) following GO terms. The qPCR analysis was performed with the mean of technical replicates for each batch analyzed using VH control and PA treatment. The three batches are shown as a black dot (VH) and as a black square (PA), and the horizontal bars at the extremes represent the standard deviation (SD) from three batches, while the middle bar corresponds to the mean relative expression, which represents the log_10_(FC). Gene expression was normalized to the *GAPDH *reference gene. Statistical analysis was based on an unpaired *T*-test with a *p-*value < 0.05 to compare PA with VH. PA, palmitic acid and VH, vehicle

The top 20 hub genes were calculated using four algorithms (MNC, degree, MCC, and DMNC) with CytoHubba. Upon intersection of the UpSet diagram, 8 common hub genes were found: *IFIT2*, *IRF1*, *XAF1*, *MX1*, *IFI35*, *STAT1*, *IFI6*, and *IFI27* (Fig. 6A and Table 1). It is worth mentioning that these 8 hub genes were significantly upregulated, and primarily comprised module 1, which was linked to inflammation network and antiviral immune response as shown in Fig. 5B. Additionally, GO BP analysis showed that genes were mainly involved in the defense response to virus and IFN signaling pathway (Fig. 6B).

**Table 1:** Hub genes are identified by intersection of four algorithms (MNC, degree, MCC, and DMNC), which are upregulated in human astrocytes associated with PA-induced inflammatory processes

Hub genes	MCODE Connectivity			PA		TIBPA		PathCards
	Module	Status	Score	log_2_FC	FDR	log_2_FC	FDR	SuperPathway
Interferon induced protein with tetratricopeptide repeats 2 (*IFIT2*)	1	Clustered	22.92	4.74	3.40E-07	5.18	7.87E-08	Immune response IFN alpha/beta signaling pathway
Interferon regulatory factor 1 (*IRF1*)	1	Clustered	22.92	2.90	5.62E-09	3.33	6.61E-10	Innate immune system
XIAP associated factor 1 (*XAF1*)	1	Clustered	22.92	2.41	6.16E-05	3.03	1.17E-06	Apoptosis and autophagy
MX dynamin like GTPase 1 (*MX1*)	1	Clustered	22.92	2.06	1.61E-06	2.43	1.70E-07	Cytokine signaling in immune system
Interferon induced protein 35 (*IFI35*)	1	Clustered	22.92	1.75	3.98E-05	1.98	5.75E-06	Cytokine signaling in immune system
Signal transducer and activator of transcription 1 (*STAT1*)	1	Clustered	23	1.15	4.03E-05	1.45	2.2E-06	IL-4 signaling pathways
Interferon alpha inducible protein 6 (*IFI6*)	1	Clustered	22.92	1.35	0.0048	1.76	0.00043	Immune response IFN alpha/beta signaling pathway
Interferon alpha inducible protein 27 (*IFI27*)	1	Clustered	22.92	1.15	0.014	1.18	0.0090	Cytokine signaling in immune system

PA, palmitic acid; and TIBPA, tibolone +palmitic acid

On the other hand, considering the relevance of these hub genes, we selected the top three to carry out the validation of results obtained by RNA-seq analysis. We included three up- and downregulated genes according to GO terms to complete this validation using qRT-PCR (Table S3). We found that *IFIT2*, *IRF1*, and *XAF1* were increased by PA in comparison to VH (*p-*value < 0.05) (Fig. 6C–E, respectively). Regarding *LMOD1*, *ACTC1*, and *RFLNA*, we detected by qRT-PCR that PA decreased their expression in contrast to VH (*p-*valu*e* < 0.05) (Fig. 6F–H, respectively). These data were correlated with those observed by RNA-seq.

## Discussion

Excess of saturated fatty acids as PA on astrocytes may trigger several disruptions associated with neuropathologies, including AD, but what determines this is poorly understood. To cope with this, we present the most comprehensive transcriptomic analysis of NHA stimulated with PA, and assess the levels of TIB protective factors in response to lipotoxic insult [18]. Our transcriptome profiling reveals that astrocytes undergo a profound transcriptional change at 2 mM PA, affecting the expression of 739 DEGs, 366 up- and 373 downregulated, whereas TIB at 10 nM does not entirely reverse PA detrimental effects. Notably, PA appears to alter the expression of miscellaneous genes associated with astrocyte defense responses by upregulating pathways associated with antiviral innate immunity, and lipid metabolism. To the best of our knowledge, we report that activation of viral response signaling pathways might be the initial molecular mechanism of astrocytes against PA toxicity, triggered mainly upon increased expression levels of *IFIT2*, *IRF1*, *XAF1*, *MX1*, *IFI35*, *STAT1*, *IFI6*, and *IFI27*.

The activation of antiviral innate immunity pathways leads to the synthesis of type I interferons (IFN) by means of Toll-like receptors (TLRs) and retinoic acid-inducible gene – I (RIG-I) [44]. Interestingly, it has been demonstrated that PA induces inflammation in astrocytes through the TLR4 [45], and other studies have suggested that this fatty acid acts as a TLR agonist [46]. These findings explain why in our bioinformatics analysis, several DEGs were associated with antiviral innate pathways. On the other hand, in the PPI analysis, the most significant BP terms associated with proteins of module 1 was the type I interferon signaling pathway, which is responsible for antiviral response in infected cells [47]. Remarkably, several studies have found that this pathway is activated in astrocytes under different experimental conditions, namely in an amyotrophic lateral sclerosis model [48], in a traumatic brain injury model [49], and in exposure to cocaine [50]. Although the type I interferon signaling pathway is important to protect the brain against viral infection-induced damage, it has been demonstrated that its upregulation can be deleterious, and it is associated with ND [51, 52]. In fact, a recent study has demonstrated that impaired type I interferon signaling activity may increase the risk of progression in pre-clinical AD [53]. Therefore, it may suggest that saturated fatty acids participate in the induction of neurodegeneration through activation of the type I interferon signaling pathway.

The second module in the PPI was associated with lipid metabolism, mainly cholesterol biosynthesis. Cholesterol is part of membranes and is a precursor of signaling molecules, impacting processes such as neurotransmission, and synaptic formation. In the brain, cholesterol should be synthesized in situ mainly by astrocytes, and its dysregulation has been related to ND, including the altered transport by *APOE* protein [54, 55]. Besides, it has also been reported that high levels of cholesterol lead to the accumulation of beta-amyloid peptides by influencing the function of *β*- and *γ*-secretase, a mechanism associated with AD [56]. Another important observation in previous studies is that PA can induce cholesterol accumulation in HepG2 cells through TLR4/MyD88/NF-κB signaling pathway by induction of *SREBP2* expression [57]. Considering the above background, our findings regarding the upregulation of genes associated with cholesterol biosynthesis, including *SREBP2* might explain the link between saturated fatty acid and ND. Moreover, our results highlight that the TLR4 receptor is key for the induction of PA effects since several genes are associated with pathways that need its activation.

In the PPI analysis, two seed proteins were identified, *MX2* and *SREBP2*. Seed proteins facilitate the understanding of pathogenic mechanisms that trigger the onset and progression of diseases [58]. Based on this concept, *MX2* is important for an antiviral response, which is induced by interferons [59]. Interestingly, similar to our results, the homolog gene in mouse *MX2* is also increased by an inflammatory stimulus [60]. Moreover, in ReN-derived astrocytes stimulated with cytokines, the protein encoded by *MX2* was upregulated [61]. Likewise, in the brain tissue of multiple sclerosis patients, *MX2* protein was detected in most cases compared to controls [62]. These data suggest a key role of this gene in inflammatory processes in the brain and specifically, in astrocytes. On the other hand, *SREBP2*, a gene involved in cholesterol biosynthesis, was upregulated by PA in NHA cells. This finding was similar by another study on mouse neuroblastoma cells containing the double mutant *AβPP69* [63], where PA induced the expression of *SREBP1 *and *SREBP2 *genes. Thus, our results are relevant because these genes confirm the effects observed in previous studies. Nevertheless, further analysis will be important to elucidate the role of these seed proteins in inflammatory processes or activation in astrocytes.

Finally, we observed that TIB could modulate the expression of the *DUSP1* gene. This gene is involved in mechanisms of dephosphorylation of MAP kinase MAPK1/ERK2 [64]. Previously, it has been demonstrated that *DUSP1* is a common gene induced by astrocytes under different stressful stimuli [5]. Therefore, it might be an interesting target for further studies about the effects of TIB on astrocyte activation attenuation. On the other hand, conversely to previous reports [18], our findings showed that TIB did not reverse the transcriptional changes induced by PA in astrocytes during the experimental conditions, despite its protective effect at the cellular level. However, the proteomic analysis identified that TIB altered the expression of proteins associated with the endoplasmic reticulum exit site and cadherin binding in comparison with PA [20]. These different results of TIB in a lipotoxic astrocytes scenario are probably due to variability in the expression pattern of estrogen receptors (ERs) [65, 66], or because the molecular responses after ER activation can be time-dependent [67]. Moreover, our results also suggest that TIB might induce non-genomic mechanisms to protect the cells similar to estrogen [68], which involves rapid responses that cannot be observed after several hours of stimulus, and this in part might explain the findings in the metabolomic analysis of NHA cells, where differences were not detected after 24 h of TIB stimulus followed by PA treatment [19].

## Conclusions

In this work, we performed the first transcriptome-wide investigation of the impact of saturated fatty acids and to what extent they respond to protective functions against inflammation in NHA. We demonstrate that at 2 mM PA astrocytes suffer an increased transcriptional alteration, which initially activates a defensive response against lipotoxicity to viral-like mechanisms. It also underscores the widespread immune-metabolic dysregulation induced by PA on astrocytes, whereby TIB was not able to fully reverse the lipotoxic impact of this saturated fatty acid. These findings may shed light on the pleiotropic effects of PA on astrocytes, in which high concentrations of this fatty acid are a risk factor for the development of different ND. Further studies are needed to elucidate the complete role of saturated fatty acids in ND, which will be relevant for testing and designing new therapeutic strategies.

## Supplementary Information

Below is the link to the electronic supplementary material.

Supplementary Figures (DOCX 2.62 MB)

Supplementary Table 1 (DOCX 12 KB)

Supplementary Table 2 (DOCX 13 KB)

Supplementary Table 3 (DOCX 15 KB)

Supplementary Table 4 (XLSX 168 KB)

Supplementary Table 5 (XLSX 50 KB)

Supplementary Table 6 (XLSX 78 KB)

## Data Availability

RNA-Seq data generated for this paper are deposited to the NCBI’s Gene Expression Omnibus (GEO) database with accession number: GSE166500.

## Data and Code Availability

Code and datasets to reproduce the figures presented as well as all analysis results are available on GitHub repository: https://github.com/alrojascr/Astrocyte-RNAseq-Analysis. Any additional information required to re-analyze the data reported in this paper is available from the lead contact upon request.
