# Supplementary material for: Palmitic Acid Upregulates Type I Interferon–Mediated Antiviral Response and Cholesterol Biosynthesis in Human Astrocytes
Source: Mol Neurobiol. 2023 May 15;60(8):4842–54. doi: 10.1007/s12035-023-03366-z (PMC10293381; doi:10.1007/s12035-023-03366-z)
Supplement: Supplementary file 1 — Supplementary Figures (DOCX 2.62 MB) [file 12035_2023_3366_MOESM1_ESM.docx]

**Palmitic Acid Upregulates Type I Interferon-Mediated Antiviral Response and Cholesterol Biosynthesis in Human Astrocytes**

Alexis Felipe Rojas-Cruz^1^, Cynthia Alexandra Martín-Jiménez^2^, Janneth González^1^, Yeimy González-Giraldo^1^, Andrés Mauricio Pinzón^3^, George E. Barreto^4^, Andrés Felipe Aristizábal-Pachón^1^*

^1^ Departamento de Nutrición y Bioquímica, Facultad de Ciencias, Pontificia Universidad Javeriana, Bogotá 110231, Colombia

^2^ Department of Neuroscience and Regenerative Medicine, Medical College of Georgia at Augusta University, Augusta, GA 30912, USA

^3^ Laboratorio de Bioinformática y Biología de Sistemas, Universidad Nacional de Colombia, Bogotá 110231, Colombia

^4^ Department of Biological Sciences, University of Limerick, V94 T9PX Limerick, Ireland

**Supplementary Figures**

**Fig. S1** Impact of PA and TIB on propidium iodide (PI) uptake by NHA cells. **(A)** Graph shows that 2 mM of PA for 24 h causes a 50% increase in PI uptake compared with control NHA cells (p < 0.0001); instead **(B)** the results of PI uptake when astrocytes are pre-treated with TIB for 24 h and then exposed to PA for 24 h, demonstrating that at 10 nM TIB attenuates 53% of cell death induced by PA (p < 0.0001). A one-way ANOVA test is used to evaluate significant differences between treatments, and data are presented as Standard Error of the Mean (SEM), using GraphPad Prism.

**Fig. S2** Statistical assessment of depth normalization of transcriptome profiling of astrocytes upon PA toxicity. **(A)** Boxplot distribution of read counts (logCPM) in each sample after normalization by Trimmed Mean of M-values (TMM) method. The center line, box extent, and dots denote the 50th percentile, 25th, and 75th percentiles, and outliers of estimates, respectively. **(B)** Principal Component Analysis (PCA) of normalized RNA-seq read counts. For each treatment group, sample distances are illustrated on the first two principal components.

**Fig. S3** Bubble plot showing significantly enriched Gene Ontology (GO) terms for the differentially expressed genes (DEGs) in astrocytes subjected to PA. **(A)** GO terms for up- and **(B)** downregulated DEGs. The y-axis represents the -log_10_ (*p-value*) and the x-axis represents the z-score. Size of the bubbles is proportional to the number of DEGs (adj. *p-value* < 0.05) assigned to the GO term.

**Fig. S4** Enriched KEGG pathways for two protein clustering modules from PPI network. **(A)** The first clustering module shows that proteins are related to antiviral and inflammatory responses, **(B)** while the second module is involved in lipid metabolism pathways. Significantly enriched KEGG analysis were carried out considering an FDR-adjusted *p-value* < 0.05 by Benjamini-Hochberg method. Circle size is proportional to the GeneRatio, while color denotes significance (orange is more significant, and purple is less significant).
