# Supplementary material for: Palmitic Acid Upregulates Type I Interferon–Mediated Antiviral Response and Cholesterol Biosynthesis in Human Astrocytes
Source: Mol Neurobiol. 2023 May 15;60(8):4842–54. doi: 10.1007/s12035-023-03366-z (PMC10293381; doi:10.1007/s12035-023-03366-z)
Supplement: Supplementary file 2 — Supplementary Table 1 (DOCX 12 KB) [file 12035_2023_3366_MOESM2_ESM.docx]

**Table S1**. General description of the 30 samples analyzed in this study

| **Organism** | **Cell line** | **Biological replicate** | **Technical replicate** | **Sex** | **Treatment** | **RIN** | **DV 200 (RNA)** | **Molecule** | **Description** |
| --- | --- | --- | --- | --- | --- | --- | --- | --- | --- |
| Homo sapiens | Normal human Astrocytes | Normal Human Astrocyte 1 Batch 0000514417 | 1 | Male | Tibolone | 10 | 87.16 | RNA with polyA selection | AstroTIB.R1A |
| Homo sapiens | Normal human Astrocytes | Normal Human Astrocyte 1 Batch 0000514417 | 2 | Male | Tibolone | 10 | 87.45 | RNA with polyA selection | AstroTIB.R1B |
| Homo sapiens | Normal human Astrocytes | Normal Human Astrocyte 1 Batch 0000514417 | 1 | Male | DMEM | 10 | 85.88 | RNA with polyA selection | AstroDMEM.R1A |
| Homo sapiens | Normal human Astrocytes | Normal Human Astrocyte 1 Batch 0000514417 | 2 | Male | DMEM | 10 | 86.06 | RNA with polyA selection | AstroDMEM.R1B |
| Homo sapiens | Normal human Astrocytes | Normal Human Astrocyte 1 Batch 0000514417 | 1 | Male | Tibolone+Palmitic Acid | 10 | 80.89 | RNA with polyA selection | AstroTIBPA.R1A |
| Homo sapiens | Normal human Astrocytes | Normal Human Astrocyte 1 Batch 0000514417 | 2 | Male | Tibolone+Palmitic Acid | 10 | 83.39 | RNA with polyA selection | AstroTIBPA.R1B |
| Homo sapiens | Normal human Astrocytes | Normal Human Astrocyte 1 Batch 0000514417 | 1 | Male | Palmitic Acid | 9.9 | 82.98 | RNA with polyA selection | AstroPA.R1A |
| Homo sapiens | Normal human Astrocytes | Normal Human Astrocyte 1 Batch 0000514417 | 2 | Male | Palmitic Acid | 9.5 | 78.4 | RNA with polyA selection | AstroPA.R1B |
| Homo sapiens | Normal human Astrocytes | Normal Human Astrocyte 1 Batch 0000514417 | 1 | Male | Vehicle | 10 | 85.4 | RNA with polyA selection | AstroVH.R1A |
| Homo sapiens | Normal human Astrocytes | Normal Human Astrocyte 1 Batch 0000514417 | 2 | Male | Vehicle | 9.8 | 80.01 | RNA with polyA selection | AstroVH.R1B |
| Homo sapiens | Normal human Astrocytes | Normal Human Astrocyte 2 Batch 0005656712 | 1 | Female | Tibolone | 9.3 | 68.85 | RNA with polyA selection | AstroTIB.R2A |
| Homo sapiens | Normal human Astrocytes | Normal Human Astrocyte 2 Batch 0005656712 | 2 | Female | Tibolone | 10 | 68.88 | RNA with polyA selection | AstroTIB.R2B |
| Homo sapiens | Normal human Astrocytes | Normal Human Astrocyte 2 Batch 0005656712 | 1 | Female | DMEM | 9.8 | 70.78 | RNA with polyA selection | AstroDMEM.R2A |
| Homo sapiens | Normal human Astrocytes | Normal Human Astrocyte 2 Batch 0005656712 | 2 | Female | DMEM | 9.6 | 69.66 | RNA with polyA selection | AstroDMEM.R2B |
| Homo sapiens | Normal human Astrocytes | Normal Human Astrocyte 2 Batch 0005656712 | 1 | Female | Tibolone+Palmitic Acid | 8.2 | 58.23 | RNA with polyA selection | AstroTIBPA.R2A |
| Homo sapiens | Normal human Astrocytes | Normal Human Astrocyte 2 Batch 0005656712 | 2 | Female | Tibolone+Palmitic Acid | 9.4 | 63.63 | RNA with polyA selection | AstroTIBPA.R2B |
| Homo sapiens | Normal human Astrocytes | Normal Human Astrocyte 2 Batch 0005656712 | 1 | Female | Palmitic Acid | 10 | 64.38 | RNA with polyA selection | AstroPA.R2A |
| Homo sapiens | Normal human Astrocytes | Normal Human Astrocyte 2 Batch 0005656712 | 2 | Female | Palmitic Acid | 9.6 | 67.2 | RNA with polyA selection | AstroPA.R2B |
| Homo sapiens | Normal human Astrocytes | Normal Human Astrocyte 2 Batch 0005656712 | 1 | Female | Vehicle | 9 | 65.84 | RNA with polyA selection | AstroVH.R2A |
| Homo sapiens | Normal human Astrocytes | Normal Human Astrocyte 2 Batch 0005656712 | 2 | Female | Vehicle | 9.7 | 67.3 | RNA with polyA selection | AstroVH.R2B |
| Homo sapiens | Normal human Astrocytes | Normal Human Astrocyte 3 Batch 0000612736 | 1 | Female | Tibolone | 9.4 | 66.77 | RNA with polyA selection | AstroTIB.R3A |
| Homo sapiens | Normal human Astrocytes | Normal Human Astrocyte 3 Batch 0000612736 | 2 | Female | Tibolone | 9.8 | 69.31 | RNA with polyA selection | AstroTIB.R3B |
| Homo sapiens | Normal human Astrocytes | Normal Human Astrocyte 3 Batch 0000612736 | 1 | Female | DMEM | 9.5 | 70.37 | RNA with polyA selection | AstroDMEM.R3A |
| Homo sapiens | Normal human Astrocytes | Normal Human Astrocyte 3 Batch 0000612736 | 2 | Female | DMEM | 9.8 | 73.57 | RNA with polyA selection | AstroDMEM.R3B |
| Homo sapiens | Normal human Astrocytes | Normal Human Astrocyte 3 Batch 0000612736 | 1 | Female | Tibolone+Palmitic Acid | 9.9 | 64.3 | RNA with polyA selection | AstroTIBPA.R3A |
| Homo sapiens | Normal human Astrocytes | Normal Human Astrocyte 3 Batch 0000612736 | 2 | Female | Tibolone+Palmitic Acid | 9.6 | 61.34 | RNA with polyA selection | AstroTIBPA.R3B |
| Homo sapiens | Normal human Astrocytes | Normal Human Astrocyte 3 Batch 0000612736 | 1 | Female | Palmitic Acid | 8.6 | 59.22 | RNA with polyA selection | AstroPA.R3A |
| Homo sapiens | Normal human Astrocytes | Normal Human Astrocyte 3 Batch 0000612736 | 2 | Female | Palmitic Acid | 9.3 | 60.44 | RNA with polyA selection | AstroPA.R3B |
| Homo sapiens | Normal human Astrocytes | Normal Human Astrocyte 3 Batch 0000612736 | 1 | Female | Vehicle | 9.1 | 60.84 | RNA with polyA selection | AstroVH.R3A |
| Homo sapiens | Normal human Astrocytes | Normal Human Astrocyte 3 Batch 0000612736 | 2 | Female | Vehicle | 9.9 | 58.62 | RNA with polyA selection | AstroVH.R3B |
