# Supplementary material for: Palmitic Acid Upregulates Type I Interferon–Mediated Antiviral Response and Cholesterol Biosynthesis in Human Astrocytes
Source: Mol Neurobiol. 2023 May 15;60(8):4842–54. doi: 10.1007/s12035-023-03366-z (PMC10293381; doi:10.1007/s12035-023-03366-z)
Supplement: Supplementary file 3 — Supplementary Table 2 (DOCX 13 KB) [file 12035_2023_3366_MOESM3_ESM.docx]

**Table S2.** rRNA databases used to map clean FasQC into rRNA and non-rRNA

| **Database** | **Domain** | **SU rRNA** | **Reference** |
| --- | --- | --- | --- |
| Rfam | Eukaryota | 5.8s | rfam-5.8s-database-id98.fasta |
| Rfam | Eukaryota | 5s | rfam-5s-database-id98.fasta |
| SILVA | Archaea | 16s | silva-arc-16s-id95.fasta |
| SILVA | Archaea | 23s | silva-arc-23s-id98.fasta |
| SILVA | Bacteria | 16s | silva-bac-16s-id90.fasta |
| SILVA | Bacteria | 23s | silva-bac-23s-id98.fasta |
| SILVA | Eukaryota | 18s | silva-euk-18s-id95.fasta |
| SILVA | Eukaryota | 28s | silva-euk-28s-id98.fasta |
