# Supplementary material for: Palmitic Acid Upregulates Type I Interferon–Mediated Antiviral Response and Cholesterol Biosynthesis in Human Astrocytes
Source: Mol Neurobiol. 2023 May 15;60(8):4842–54. doi: 10.1007/s12035-023-03366-z (PMC10293381; doi:10.1007/s12035-023-03366-z)
Supplement: Supplementary file 4 — Supplementary Table 3 (DOCX 15 KB) [file 12035_2023_3366_MOESM4_ESM.docx]

**Table S3.** Primer sequence and annealing temperature (Ta)

| **Gene** | **Forward primer** | **Reverse primer** | **Ta** |
| --- | --- | --- | --- |
| *LMOD1* | GAAGAACTCCCGTGACCAGCTA | AGCCTGGTCCTACTGAAGCAGT | 60 |
| *ACTC1* | GAAGAACTCCCGTGACCAGCTA | AGCCTGGTCCTACTGAAGCAGT | 60 |
| *RFLNA* | GCAACTCTGAGGTCAAGTACGC | GCAGTTGGGTGCTGCCACGAT | 60 |
| *IFIT2* | GGAGCAGATTCTGAGGCTTTGC | GGATGAGGCTTCCAGACTCCAA | 65 |
| *IRF1* | GAGGAGGTGAAAGACCAGAGCA | TAGCATCTCGGCTGGACTTCGA | 60 |
| *XAF1* | CCTCCATGAGGCTTACTGCCTG | GAAACTCCAGCGAGGACTTCTG | 65 |
| *GAPDH* | CATCAATGGAAATCCCATCA | TTCTCCATGGTGGTGAAGAC | 60 |
